# Multiparametric Prostate MRI Accuracy of Prostate Imaging Reporting and Data System (v2.1) Scores 4 and 5: The Influence of Image Quality According to the Prostate Imaging Quality Score

**DOI:** 10.3390/jcm13133785

**Published:** 2024-06-27

**Authors:** Andrea Fuschi, Paolo Pietro Suraci, Antonio Luigi Pastore, Yazan Al Salhi, Paola Capodiferro, Silvio Scalzo, Onofrio Antonio Rera, Fabio Maria Valenzi, Michele Di Dio, Pierluigi Russo, Mohammad Talal Al-Zubi, Saddam Al Demour, Samer Fathi Al-Rawashdah, Giorgio Mazzon, Davide Bellini, Iacopo Carbone, Vincenzo Petrozza, Giorgio Bozzini, Alessandro Zucchi, Matteo Pacini, Giorgia Tema, Cosimo De Nunzio, Antonio Carbone, Marco Rengo

**Affiliations:** 1 Unit of Urology, Department of Medical-Surgical Sciences and Biotechnologies, I.C.O.T. Hospital, University of Rome Sapienza, Via F. Faggiana 1668, 04100 Latina, Italy; andrea.fuschi@uniroma1.it (A.F.); spaolopietro@gmail.com (P.P.S.); yazan.alsalhi@uniroma1.it (Y.A.S.); silvioscalzo@hotmail.it (S.S.); antoniorera94@gmail.com (O.A.R.); fabiovalenzi@gmail.com (F.M.V.); antonio.carbone@uniroma1.it (A.C.); 2 Department of Radiological, Oncological and Pathological Sciences, Academic Diagnostic, Imaging Division, I.C.O.T. Hospital, University of Rome Sapienza, Via F. Faggiana 1668, 04100 Latina, Italy; paola.capodiferro@uniroma1.it (P.C.); davide.bellini@uniroma1.it (D.B.) iacopo.carbone@uniroma1.it (I.C.); marco.rengo@uniroma1.it (M.R.); 3 Division of Urology, Department of Surgery, Annunziata Hospital, 87100 Cosenza, Italy; m.didio@aocs.it; 4 Università Cattolica del Sacro Cuore, Largo Francesco Vito 1, 00168 Rome, Italy; pierluigi.russo@guest.policlinicogemelli.it; 5 Urology Unit, Fondazione Policlinico A. Gemelli IRCCS, Largo Agostino Gemelli 8, 00168 Rome, Italy; 6 Department of Surgery, Division of Urology, School of Medicine, Yarmouk University, Irbid 21110, Jordan; mzubi@yu.edu.jo; 7 Department of Special Surgery, Division of Urology, School of Medicine, The University of Jordan, Amman 11972, Jordan; saldemour@ju.edu.jo; 8 Department of Special Surgery, Urology Unit, School of Medicine, Mutah University, Karak 61710, Jordan; samer.rawashdah@gmail.com; 9 Institute of Urology, University College Hospital, London NW12BU, UK; giorgiomazzon83@gmail.com; 10 Pathology Unit, Department of Medical-Surgical Sciences and Biotechnologies, I.C.O.T. Hospital, University of Rome Sapienza, Via F. Faggiana 1668, 04100 Latina, Italy; vincenzo.petrozza@uniroma1.it; 11 Division of Urology, Sant’Anna Hospital, San Fermo della Battaglia, 22042 Como, Italy; gioboz@yahoo.it; 12 Department of Urology, University of Pisa, 56126 Pisa, Italy; alessandro.zucchi@unipi.it (A.Z.); matteopacini93@gmail.com (M.P.); 13 Department of Urology, Sapienza University of Rome, Sant’Andrea Hospital, Via di Grottarossa 1035-1039, 00189 Rome, Italy; giorgia.tema@uniroma1.it (G.T.); cosimo.denunzio@uniroma1.it (C.D.N.)

**Keywords:** mpMRI, PI-RADS, PI-QUAL, prostate biopsy, prostate cancer

## Abstract

**Purpose:** The accuracy of multiparametric magnetic resonance imaging (mpMRI) heavily relies on image quality, as evidenced by the evolution of the prostate imaging quality (PI-QUAL) scoring system for the evaluation of clinically significant prostate cancer (csPC). This study aims to evaluate the impact of PI-QUAL scores in detecting csPC within PI-RADS 4 and 5 lesions. **Methods:** We retrospectively selected from our database all mpMRI performed from January 2019 to March 2022. Inclusion criteria were as follows: (1) mpMRI acquired in our institution according to the technical requirements from the PI-RADS (v2.1) guidelines; (2) single lesion scored as PI-RADS (v2.1) 4 or 5; (3) MRI-TBx performed in our institution; (4) complete histology report; and (5) complete clinical record. **Results:** A total of 257 male patients, mean age 70.42 ± 7.6 years, with a single PI-RADS 4 or 5 lesion undergoing MRI-targeted biopsy, were retrospectively studied. Of these, 61.5% were PI-RADS 4, and 38.5% were PI-RADS 5, with 84% confirming neoplastic cells. In high-quality image lesions (PI-QUAL ≥ 4), all PI-RADS 5 lesions were accurately identified as positive at the final histological examination (100% of CDR). For PI-RADS 4 lesions, 37 (23%) were negative, resulting in a cancer detection rate of 77% (95% CI: 67.51–84.83). **Conclusions:** The accuracy of mpMRI, independently of the PI-RADS score, progressively decreased according to the decreasing PI-QUAL score. These findings emphasize the crucial role of the PI-QUAL scoring system in evaluating PI-RADS 4 and 5 lesions, influencing mpMRI accuracy.

## 1. Purpose

Multiparametric magnetic resonance imaging (mpMRI) is a noninvasive exam widely recommended for the detection and stratification of clinically significant prostate cancer (csPCa) [1].

The MRI-targeted biopsy (MRI-TBx), meant as the fusion between mpMRI and transrectal ultrasound, has been demonstrated to significantly outperform systematic biopsy for the detection of prostate cancer (PCa) [2,3].

The standardization of the image acquisition technique and interpretation of mpRMI is codified by the Prostate Imaging Reporting and Data System (PI-RADS) [4]. The latest PI-RADS 2.1 version has been demonstrated to outperform previous versions, reaching a sensitivity of 87% (95% CI 82–91%) and a specificity of 74% (95% CI 63–82%), with a progressive increase in the cancer detection rate (CDR) as the score increases from 1 to 5 (from 0.03 to 0.97) [5,6]. However, there is increasing evidence that the performances of mpMRI is significantly influenced by image quality [7,8]. For this reason, the prostate imaging quality (PI-QUAL) scoring system was developed based on both objective and subjective criteria, mainly influenced by the patient’s characteristics [9]. Since its introduction, the PI-QUAL scoring system has demonstrated good reproducibility and indicated that poor image quality is associated with a higher degree of uncertainty and lower accuracy [10,11,12].

Furthermore, a greater impact of the PI-QUAL scoring system on the classification of indeterminate lesions (PI-RADS 3) was recorded. It has been demonstrated that as the image quality increases, fewer lesions are classified as indeterminate and thus referred for biopsy [10,13]. Moreover, considering the high negative predictive value of PI-RADS 1 and 2 (DFS 99.6% after 3 years and 94.1% after 6 years), independently of the image quality [14], there is still limited evidence on the impact of the PI-QUAL scoring system on the accuracy of PI-RADS 4 and 5.

The aim of the present study is to evaluate the impact of image quality, according to the PI-QUAL scoring system, on the ability to account for csPCa for PI-RADS 4 (csPCa likely to be present) and 5 (csPCa highly likely to be present), using the results of MRI-TBx as reference standard.

## 2. Materials and Methods

We retrospectively selected from our database all mpMRI performed from January 2019 to March 2022. Inclusion criteria were as follows: (1) mpMRI acquired in our institution according to the technical requirements from the PI-RADS (v2.1) guidelines; (2) single PI-RADS 4 or 5 lesion; (3) MRI-TBx performed in our institution; and (4) complete histology and clinical report.

All mpMRI were acquired on a 1.5 Tesla scanner (MAGNETOM ESSENZA, Siemens Healthcare, Erlangen, Germany) using a phased-array coil in a supine position (Table 1).

All datasets were acquired according to the PI-RADS (v2.1) guidelines.

All patients received a fixed dose of contrast media (Gd-DOTA, DOTAREM^®^ 0.5 mmol/mL, Guerbet, Roissy CdG Cedex, France) using a fixed dose of 0.1 mmol/kg of body weight.

Reports were created by two expert radiologists at our institution according to the PI-RADS (v2.1) guidelines. Another two radiologists independently analyzed all datasets, evaluating the image quality according to the PI-QUAL score.

All prostate lesions scored as PI-RADS 4 or 5 underwent prostate biopsy.

All biopsies were performed by an expert urologist using an MRI-TBx fusion device (Trinity^®^ ultrasound system, KOELIS, Meylan, France).

**Figure 1:**
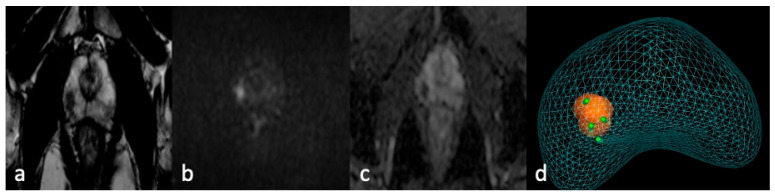
MRI-TBx procedure. Apex right PZpl prostate lesion at mpMRI (PI-RADS 4): (**a**) MRI-T2 image; (**b**) MRI-DWI image; (**c**) MRI-ADC image; (**d**) MRI-targeted biopsy report.

Before the procedure, mpMRIs were evaluated using software (ProMap Lite™ (Version 2024), KOELIS, Meylan, France) dedicated to 3D lesion semiautomatic segmentation on high-resolution T2-weighted images (Figure 1).

Trans-perineal prostate biopsy was performed according to the European Association of Urology guidelines using a 16G needle and real-time synchronization of MR and US images.

An expert uropathologist reviewed all the specimens using the Gleason score (ISUP grade group classification system) [4]. A Gleason score ≥ 6 was considered positive for PCa.

All continuous variables were expressed as mean ± standard deviation (SD) and categorical variables as counts and percentages.

Differences between categorical variables, like PI-QUAL and PI-RADS categories, and the biopsy result (positive or negative) expressed as an absolute value or a percentage of CDR, were ruled out with a chi-square test.

The inter-reader agreement for PI-QUAL scoring was evaluated by means of weighted Cohen’s kappa (κ) analysis.

Agreement was interpreted according to the following criteria: κ > 0.81: excellent agreement; κ = 0.61–0.80: good agreement; κ = 0.41–0.60: moderate agreement; κ = 0.21–0.40: fair agreement; κ < 0.20: poor agreement.

*p*-value < 0.05 was considered statistically significant.

Statistical analyses were performed with Statistical Package for Social Sciences (SPSS) software (version 25.0; SPSS Inc., Chicago, IL, USA), GraphPad prism 10 (version 10.1.0, GraphPad Software^®^, La Jolla, CA, USA), and MedCalc (version 12.5, MedCalc Software^®^, Ostend, Belgium).

## 3. Results

From a total of 786 mpMRI exams, all PI-RADS scores < 4 (374 patients) and multifocal prostate lesions (116 patients) were excluded. Among the 296 remaining patients, a total of 39 were excluded due to an absence of fusion biopsy (30), incomplete histology report (4), and incomplete clinical records (5).

The final study population consisted of 257 patients (mean age 70.42 ± 7.6 years; range 50–85 years) (Table 2).

Fewer lesions were localized in the transitional zone than in the peripheral zone (15.5% vs. 84.5%; *p* < 0.0001), 158 lesions (61.5%) were scored as PI-RADS 4, and 99 (38.5%) were scored as PI-RADS 5.

Total PSA was significantly higher in PI-RADS 5 compared to PI-RADS 4 (33.6 ng/mL ± 43.8 vs. 8.87 ng/mL ± 7.4; *p*-value: 0.002), as well as in PSA density (0.52 ng/mL^2^ ± 0.62 vs. 0.18 ng/mL^2^ ± 0.2; *p*-value: 0.009). No statistically significant differences were observed for PSA ratio, Free PSA, and prostate volume between PI-RADS 4 and 5.

PCa was confirmed in 216 (84%) lesions. The true positive ratio was significantly higher in PI-RADS 5 compared to PI-RADS 4 (95% vs. 76.6; *p*-value < 0.001).

The overall image quality (Table 3) was high (PI-QUAL ≥ 4) in 77% of the population (198 patients), sufficient (PI-QUAL 3) in 15.9% (41 patients), and below the minimum standard (PI-QUAL ≤ 2) in 7.1% (18 patients).

The distribution of PI-QUAL categories was not significantly different between PI-RADS 4 and 5 (*p*-value: 0.186).

Image quality was influenced by the presence of artifacts determined by the patients’ characteristics. T2w images were all at least acceptable; for movement artifacts, radial k-space sampling was used or the sequence was repeated; for metal artifacts, the encoding phase direction was modified. DWI/ADC images were impaired in 78 patients (30.3% of the entire population). The cause of the impairment was determined by hip prosthesis (18; 23.1%), other metal devices (22; 28.2%), rectal air (23; 29.5%), and movement artifacts (15; 19.2%). DCE images were impaired in 95 patients (36.9% of the entire population). The cause of the impairment was determined by hip prostheses (18; 18.9%), other metal devices (28; 29.5%), rectal air (15; 15.8%), and movement artifacts (34; 35.8%). All PI-QUAL 2 (18) were determined by the presence of hip prostheses, which impaired the quality of both DWI and DCE. However, 25 patients with hip prostheses were enrolled in the study population, but 7 prostheses did not significantly impair the image quality due to the technical characteristics of the device.

The CDR of mpMRI progressively decreased with the decreasing of the PI-QUAL score independently of the PI-RADS score. The CDR for PI-QUAL 5 was 90.91% (95% CI: 81.26–96.59); for PI-QUAL 4, it was 87.12% (95% CI: 80.18–92.32); for PI-QUAL 3, it was 73.17% (95% CI: 57.05–85.78); and for PI-QUAL 2, it was 61.11% (95% CI: 35.75–82.70).

Negative biopsies were significantly higher for PI-RADS 4 lesions than for PI-RADS 5 (23.4% vs. 4%; *p*-value < 0.001) (Table 4).

All PI-RADS 5 with high image quality (75 lesions) were positive at pathology (CDR 100%), while 23% (23 lesions) of PI-RADS 4 with high image quality were negative at pathology (CDR 77%; 95% CI: 67.51–84.83). The CDR of PI-RADS 4 was significantly lower compared to PI-RADS 5 for both PI-QUAL 5 and 4 (*p*-value: 0.038 and 0.001).

When image quality was sufficient or below the minimum standard, a decrease in the CDR was observed (CDR 69.49%; 95% CI: 56.13–80.81), while the CDR for PI-RADS 4 and 5 was not significantly different (*p*-value: 0.06 and 0.205).

The overall inter-reader agreement for PI-QUAL scoring, considering all PI-QUAL categories, was good (k: 0.73; 95% CI: 0.59 to 0.91). A good agreement was also observed after clustering the PI-QUAL categories as follows: PI-QUAL ≥ 4: high image quality (k: 0.75; 95% CI: 0.63 to 0.95); PI-QUAL 3: sufficient image quality (k: 0.72; 95% CI: 0.57 to 0.89); and PI-QUAL ≤ 2: poor image quality (k: 0.68; 95% CI: 0.48 to 0.82).

## 4. Discussion

The results confirmed the importance of mpMRI image quality for PI-RADS 4 and 5 lesions. According to the latest metanalysis, the CDR for PI-RADS 4 and 5 is 70% (95% CI: 61–79%) and 97% (95% CI: 92–99%), respectively, independently of image quality [6]. Our results are in line with these outcomes since the overall CDR was 76.6% and 96% for PI-RADS 4 and 5, respectively. When CDR was stratified according to the PI-QUAL system, a heterogeneous scenario was observed. In PI-RADS 5 lesions, the CDR reached 100% when image quality was high (PI-QUAL ≥ 4), while it decreased to 92.3% for sufficient image quality (PI-QUAL 3) and 72.7% for image quality below the minimum standard (PI-QUAL ≤ 2). The same trend was observed for PI-RADS 4 lesions, where CDR reached 82.6% in high image quality, while it decreased to 64.3% for sufficient image quality and 42.8% for image quality below the minimum standard. That CDR reduces with the reduction of the PI-QUAL score was mainly due to the difficulty involved in contouring the prostate. These results are in line with the recently published data by Brembilla et al., confirming a decrease in the CDR from high image quality (PI-QUAL 4–5) to lower image quality (PI-QUAL 2–3) [13].

The results also confirmed the good reproducibility of the PI-QUAL scoring system. A good (k: 0.73) overall agreement was observed for all PI-QUAL categories, as well as for grouped categories (≥4, 3 and ≤2; k: 0.75, 0.72, and 0.78, respectively), in line with previous publications [11,15].

Furthermore, despite all exams being conducted in strict accordance with the PI-RADS 2.1 recommendations, the image quality was only sufficient or below the minimum standard in 15.9% and 7.1% of the exams, respectively. Thus, patient factors causing artifacts can impair image quality and the accuracy of mpMRI.

In fact, significant artifacts can lead to misreading potential target lesions. Poor image quality may not solely result from artifacts but could also stem from inherent MRI characteristics like magnet strength, hardware, and scanning sequences. Consequently, centers experiencing consistently low image quality may yield poorer results across various PIRADS categories, underscoring the importance of leveraging the PI-QUAL score to enhance image quality.

The first limitation of the study can be considered the use of a specialized radiologist in an academic environment since our results may not be reproducible when general radiologists are involved. Secondly, this was a retrospective study and may therefore have intrinsic limitations such as selection bias.

A third limitation may be the absence of a sub-analysis considering the location of the lesion. We did not perform a separate analysis for prostate lesions location since the number of lesions in the transitional zone was significantly lower than those in the peripheral zone. This would have impaired the statistical significance of such a sub-analysis. Finally, the use of new tools [16] for the semiautomatic image quality assessment would have improved the robustness of our results.

In conclusion, the results of the present study confirmed the pivotal role of the PI-QUAL scoring system in the assessment of PI-RADS 4 and 5 lesions, where csPCa is likely or highly likely to be present, and the influence of image quality on the accuracy of mpMRI.

## Figures and Tables

**Table 1:** mpMRI protocol details.

	T2W	T2W	DWI	DCE
Sequence type	TSE	TSE	EPI	GRE
Orientation	Axial	Sagittal	Axial	Axial
TR (ms)	6350	6000	4800	4.3
TE (ms)	117	118	76	1.7
FOV (mm)	200 × 200	200 × 200	220 × 220	220 × 220
Matrix size (PhxFq, mm)	320 × 524	224 × 320	124 × 124	154 × 192
In-plane dimension (mm)	0.62 × 0.38	0.89 × 0.62	1.77 × 1.77	1.42 × 1.14
Slice thickness (mm)	2	3	3	2
Slice gap (mm)	0	0	0	0
b-values (s/mm^2^)	-	-	50, 1000, 1400	-
Temporal Resolution (s)	-	-	-	≤15
Total acquisition (min)	-	-	-	2

TSE: turbo spin echo; EPI: echo planar imaging; GRE: gradient echo; TE: echo time; FOV: field of view.

**Table 2:** Study population characteristics.

	All	PI-RADS 4	PI-RADS 5	*p*-Value ^2^
Total PSA (ng/mL)	17.86 (±29.2)	8.87 (±7.4)	33.6 (±43.8)	0.002
Free PSA (ng/mL)	2.43 (±3.2)	2.3 (±3.6)	2.7 (±2.8)	0.773
PSA ratio (%)	18.41 (±10.5)	18.4 (±10.5)	18.4 (±11.2)	0.996
PSA density (ng/mL ^2^)	0.31 (±0.43)	0.18 (±0.2)	0.52 (±0.62)	0.009
Prostate volume (mL) ^1^	58.74 (±31.8)	61.05 (±32.5)	54.7 (±31.2)	0.525

^1^ calculated on MRI. ^2^ *t* test.

**Table 3:** Image quality and PI-QUAL score.

	All Patients	PI-RADS 4	PI-RADS 5	*p*-Value
All	257	158 (61.5%)	99 (38.5%)	0.186
PI-QUAL 5	66 (25.7%)	40 (25.3%)	26 (26.3%)	
PI-QUAL 4	132 (51.3%)	83 (52.5%)	49 (49.4%)	
PI-QUAL 3	41 (15.9%)	28 (17.7%)	13 (13.2%)	
PI-QUAL 2	18 (7.1%)	7 (4.5%)	11 (11.1%)	
PI-QUAL 1	0 (0%)	0 (0%)	0 (0%)	
Positive biopsy	216 (84%)	121 (76.6%)	95 (96%)	<0.001

**Table 4:** PI-QUAL score distribution in positive (CDR) and negative (false positive) biopsies.

	Positive Biopsy		Negative Biopsy		
	PI-RADS 4	PI-RADS 5	PI-RADS 4	PI-RADS 5	*p*-Value
All	121 (76.6%)	95 (96%)	37 (23.4%)	4 (4%)	<0.001
PI-QUAL 5	34 (85%)	26 (100%)	6 (15%)	0 (0%)	0.038
PI-QUAL 4	66 (79.5%)	49 (100%)	17 (20.5%)	0 (0%)	0.001
PI-QUAL 3	18 (64.3%)	12 (92.3%)	10 (35.7%)	1 (7.7%)	0.06
PI-QUAL 2	3 (42.8%)	8 (72.7%)	4 (57.2%)	3 (27.3%)	0.205
PI-QUAL 1	0 (0%)	0 (0%)	0 (0%)	0 (0%)	

## Data Availability

The data that support the findings of this study are available on request from the corresponding author, [ALP]. The data are not publicly available due to privacy restrictions, containing informations that could compromise the privacy of research participants.
